# Cropland changes in times of conflict, reconstruction, and economic development in Iraqi Kurdistan

**DOI:** 10.1007/s13280-015-0686-0

**Published:** 2015-07-23

**Authors:** Lina Eklund, Andreas Persson, Petter Pilesjö

**Affiliations:** Center for Middle Eastern Studies, Lund University, Finngatan 16, 22361 Lund, Sweden; Department of Earth and Ecosystem Sciences, Lund University, Sölvegatan 12, 22362 Lund, Sweden; GIS Centre, Lund University, Sölvegatan 10, 22362 Lund, Sweden

**Keywords:** Agriculture, Conflict, Crop phenology, Iraqi Kurdistan, NDVI

## Abstract

The destruction of land and forced migration during the *Anfal* attacks against the Kurds in Iraq in the late 1980s has been reported to have severe consequences for agricultural development. A reconstruction program to aid people in returning to their lands was launched in 1991. To assess the agricultural situation in the Duhok governorate during the pre-*Anfal* (A), post-*Anfal* (B), reconstruction (C), and present (D) periods, we mapped winter crops by focusing on inter-annual variability in vegetation greenness, using satellite images. The results indicate a decrease in cultivated area between period A and B, and a small increase between period B and C. This supports reports of a decline in cultivated area related to the *Anfal* campaign, and indicates increased activity during the reconstruction program. Period D showed a potential recovery with a cropland area similar to period A.

## Introduction

Since the development of sedentary agriculture some 10 000 years bp humans have secured food provision and other necessities by establishing and expanding croplands at the expense of other land covers, e.g., forests (Roberts 1998). These changes have been even more intensive during the last three centuries, with strong increases in both cropland and grassland areas (Goldewijk 2001). Although cropland may be expanding globally, some areas have seen reversed trends, which may be attributable to socio-economic and demographic factors.

Changes to land systems are either caused by slow changes (often demographic or economic development) or drastic shocks, such as sudden socio-economic or political changes, or conflicts (Baumann et al. 2014). Kuemmerle et al. (2009), for example, assessed the impacts on land use after the breakdown of socialism in Romania using Landsat satellite imagery. They found that the change from plan economy to market economy, land reforms, and demographic changes were likely behind a 21 % decline in farmland. Gibson et al. (2012) demonstrated, using Landsat imagery, how international trade sanctions in the 1990s led to agricultural extensification in an area surrounding Baghdad, Iraq. Armed conflicts have been shown to cause widespread land cover changes through migration and land abandonment, in some cases causing reduction of farmland and increased forest cover (Witmer 2008; Stevens et al. 2011; Gorsevski et al. 2012). Concurrently, Baumann et al. (2014) found high farmland abandonment rates in the conflict areas of the 1991–1994 Nagorno-Karabakh conflict between Armenia and Azerbaijan. An assessment on the effects of drought and war on agriculture in Afghanistan, during 1982–2001 found that war and civil conflicts can cause changes in vegetation characteristics similar to the effects of drought (de Beurs and Henebry 2008).

During the early 1980s, Iraq was one of the countries in the Middle East with the highest per capita food availability due to high agricultural production, especially in the northern Kurdish governorates, which accounted for 25–30 % of the total Iraqi production (WFP Iraq—North Coordination Office 2001). Since then several political events have been reported to impede the agricultural development in Iraqi Kurdistan, leading to a strong dependence on food imports (Human Rights Watch 1993; WFP Iraq—North Coordination Office 2001; Meyer and Califano 2006; Mubareka and Ehrlich 2010; Hardi 2011). The *Anfal* campaign was a series of attacks carried out by the Iraqi regime against the Kurdish population in the northern governorates between 1987 and 1988. A satellite based study by Mubareka and Ehrlich (2010) focusing on land-use changes in two areas in Iraqi Kurdistan showed that large areas of cultivated land (between 64 and 83 %) in the Jafati Valley (Sulaymaniyah governorate) were converted into grasslands after the *Anfal* campaign. The southern rim of the Duhok governorate did, however, not show any changes attributable to the *Anfal* attacks. This latter result contradicts other reports of the effects on agriculture (Human Rights Watch 1993; Hardi 2011), which motivate further analyses covering a larger area.

This paper aims at quantifying the changes in cultivated land in connection to the *Anfal* campaign, the reconstruction program, and the economic development in the Duhok governorate. We focus on winter crops, mainly because winter crops are the most common crop in the region and wheat is a major food staple in the Iraqi diet, and also because winter crops have a clear phenological cycle. The results are discussed in the context of the Kurdish Region in Iraq’s history since the 1980s.

## Materials and methods

### Study area

**Fig. 1:**
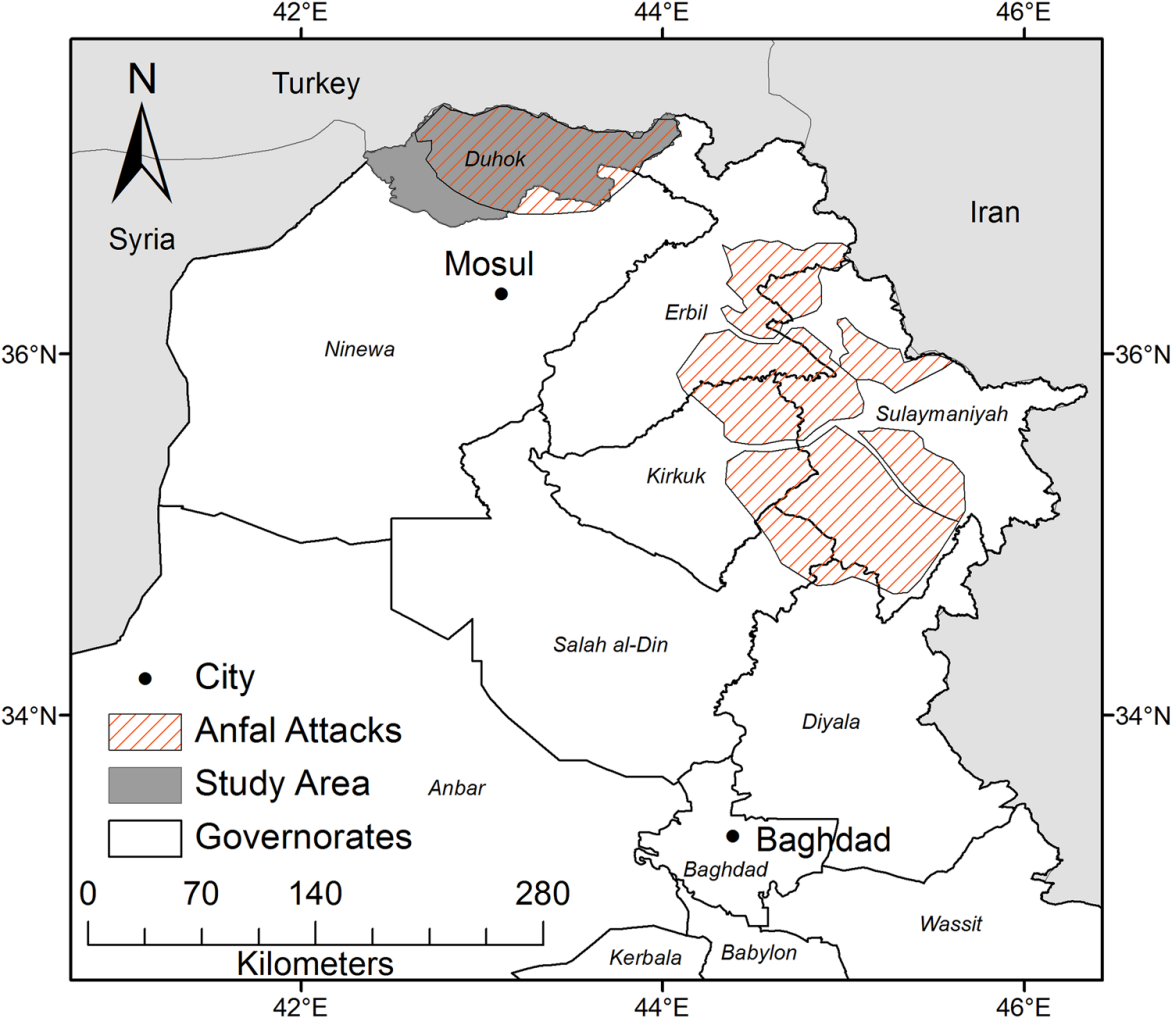
The study area location in northern Iraq and areas targeted by the *Anfal* campaign

The Duhok governorate belongs to the Kurdistan Region of Iraq (KR-I), which is a semi-autonomous area consisting of three governorates (provinces): Erbil, Sulaymaniyah, and Duhok (Fig. 1). The Duhok governorate has an area of approximately 6600 km^2^ and had an estimated population of 1.2 million people in 2009 (Kurdistan Region Statistics Office (KRSO) 2014). Since the fall of Saddam Hussein in 2003 the KR-I experienced a rapid urban development as an effect of oil revenues and increased foreign investments.

The climate of northern Iraq is characterized by high precipitation (annual mean 700 mm) due to the orographic heaving effect caused by the mountains (Trigo et al. 2010). In the plains, the main agricultural production is cereals, such as barley and wheat, which are winter crops harvested around June (Food and Agriculture Organization of the United Nations (FAO) 2004). Fallow practices include letting fields rest or changing crop type every second year.

### Agriculture since the 1970s

Social, political, and economic processes have shaped peoples relation to, and reliance on, natural resources in the KR-I. The Iraqi government under Saddam Hussein began destroying Kurdish villages in the 1970s, causing large-scale displacement of the Kurdish rural population (Barwari 2013). Between 1987 and 1989 the Iraqi government carried out a genocidal campaign known as *Al*-*Anfal* that included chemical attacks, executions, kidnappings of tens of thousands civilians, and the destruction of around 2000 villages and their agricultural land (Human Rights Watch 1993). The *Anfal* campaign consisted of eight attacks carried out in areas where the Kurdish militia (Peshmerga) was particularly influential. The first seven attacks targeted areas in the Erbil and Sulaymaniyah governorates, but the final attack was against the Duhok governorate between August 25 and September 6 1988 (Fig. 1). This period of insecurity caused a change in human activity that resulted in a conversion of agricultural land into grasslands and deforestation in the mountains (Mubareka and Ehrlich 2010).

Between 1991 and 2003, Iraq was sanctioned by the international community because of their invasion of Kuwait, which reduced the country’s purchasing power and also led to further decline in agricultural production due to lack of infrastructure and availability of agricultural input products (WFP Iraq—North Coordination Office 2001; Barwari 2013). Furthermore, after becoming autonomous in 1991, the KR-I was sanctioned by the Iraqi Government and all administrative and financial support to the region was withdrawn (Kurdistan Regional Government (KRG) 2008.

In the late 1990s, the United Nation’s Oil-for-Food programme was implemented as a way to trade oil with Iraq despite the international sanctions (Meyer and Califano 2006). Instead of paying money to the government that might be spent on the military, 2 million of the Iraqi people were provided with aid baskets, including food, medicine, and other humanitarian assets, in exchange for oil. The program lasted from 1997 until 2003 and became the major source of food in the Duhok governorate (Meyer and Califano 2006). The agricultural production in Iraq declined further and people’s lifestyle changed from being producers to consumers (WFP Iraq—North Coordination Office 2001; Food and Agriculture Organization of the United Nations (FAO) 2004). In a survey carried out in 2001 by the Food and Agricultural Organization, it was discovered that 38 % of all rural incomes in the Duhok governorate came from agriculture, while 62 % came from non-agricultural activities (Food and Agriculture Organization of the United Nations (FAO) 2004). The U.S. invasion in 2003, that led to the fall of the Iraqi regime, opened up for a new period in the KR-I. The region became safer and was no longer sanctioned, which allowed for foreign investments, a rapid economic development, and thereby increased imports. In 2010, the KR-I imported large amounts of its fruits, vegetables, and poultry from nearby countries such as Syria, Iran, and Turkey (UNDP 2010).

In an assessment of the drought that struck the KR-I between 2007 and 2009, Eklund and Seaquist (2015) found that 66 % of the Duhok governorate had experienced negative vegetation anomalies, but that only the people involved with agriculture had reported drought as problematic, indicating a lowered socio-economic susceptibility to drought due to the low dependence on agriculture.

### Migration, urbanization, and land abandonment

The conflicts between the Iraqi regime and the Kurdish population have been major causes of displacement and migration, both within and from Iraq (Eklund and Pilesjö 2012). Thousands of people were displaced during a village destruction campaign in the 1970s and displacement continued during the *Anfal* campaign (Human Rights Watch 1993; Barwari 2013). Almost 2 million Kurds fled to Iran or Turkey in March 1991 when targeted by another military campaign, after Kurdish resistance had launched a rebellion against the Iraqi Government (Barwari 2013).

A village reconstruction program was carried out between 1991 and 2003, led by the Kurdistan Regional Government (KRG) and various UN organizations with the intention of aiding people to return and reclaim their lands by restoring their livelihoods (Barwari 2013). The program included construction of houses, schools, health centers, water and sewage systems, roads, bridges, and facilities for support of agricultural and community activities, assisting over 800 000 displaced people returning to 4000 villages and towns in the KR-I.

A detailed survey of the 2000–2010 migration patterns in the Duhok governorate found that the majority of the migrating households did so for economic reasons (Eklund and Pilesjö 2012). Migration for family reasons, or marriage, was also common, as was security migration during certain periods. Eklund and Pilesjö (2012) found that more than a third (36.7 %) of the household migration patterns had been directed from urban to rural areas, indicating a “counterurbanization” trend. A field visit in 2013 revealed that this migration pattern represented return migration to villages that had been destroyed or abandoned in the 1970s or during the *Anfal* campaign. The motivations for returning to their “family village” were either security or housing issues in the city. Migration patterns in Duhok governorate since 2011 are dominated by incoming refugees from Syria and from areas targeted by the Islamic State (IS) in 2014 (Kurdistan Regional Government (KRG) 2012; IOM Iraq 2014).

### Data and methods

#### Phenology

This analysis is based on the difference in vegetation greenness of cropland between spring and summer, i.e., the periods just before and after harvest (Lenney et al. 1996; de Beurs and Henebry 2008; Pittman et al. 2010; Gibson et al. 2012). Winter crops, such as wheat and barley, are harvested in early summer and show a rapid decrease in greenness after June. Normalized Difference Vegetation Index (NDVI) can be used as a measure of vegetation greenness, where high NDVI values represent high vegetation density (Carlson and Ripley 1997).

#### Satellite data

This assessment uses Landsat data (Path: 170, Row: 034) from four periods: A (1984–1987), B (1989–1991), C (1998–2002), and D (2011–2014) (Tables 1, 2), processed with two different atmospheric correction methods. 44 scenes were downloaded as preprocessed surface reflectance images (based on the LEDAPS algorithm) from Landsat Climate Data Record recorded by the Landsat 4, 5 (Thematic Mapper, TM) and 8 (Operational Land Imager) satellites (United States Geological Survey 2014). Three unprocessed images from Landsat 5 (TM) were acquired from the United States Geological Service’s Earth Explorer and were processed using radiometric correction and Dark Object Subtraction (DOS) using the DOS3 function in the i.landsat.toar module in GRASS (see Song et al. 2001; Tizado 2014). The differences between DOS3 and LEDAPS were assessed using a scene for which both preprocessed and unprocessed data were available, and DOS3 was found to be a comparable alternative to LEDAPS, with a mean difference in NDVI of 0.03.

**Table 1:** Description of the four periods used in the analysis

Period	Characteristics	Years
A	The years before the Anfal genocide	1984–1987
B	The years after the Anfal genocide	1989–1991
	Before reconstruction had begun	
C	The final years of the reconstruction program (1991–2003)	1998–2002
	Oil-for-Food Programme (1997–2003)	
	International and national sanctions (1991–2003)	
D	After the fall of the Iraqi regime	2011–2014
	Rapid economic development	
	No longer sanctioned

**Table 2:** Years and Julian dates for the land satellite images used in the analysis, sorted on spring and summer periods. Underlined dates were used in the segmentation

Pre-*Anfal* (A)	Post-*Anfal* (B)	Reconstr. (C)	Present (D)
Spring			
1984-158	1989-35	1998-100	2011-168
1985-160	1990-86	1998-164	2013-109
1986-115	1990-134	1999-71	2013-125
1986-163	1990-166	2000-154	2013-157
1987-150	1991-129	2001-116	2014-64
		2001-148	2014-80
		2001-164	2014-96
		2002-151	2014-112
			2014-144
			2014-160
Summer			
1984-222	1989-179	1999-231	2011-184
1986-179	1989-195	2000-234	2011-232
1987-198	1989-211	2000-250	2012-179
1987-246	1990-190	2002-231	2013-173
			2013-189
			2013-237
			2014-176

In order to get a full cover of the study area, images with a low cloud cover over the study area, taken during spring/pre-harvest time (March–June) and during summer/post-harvest time (July–September), were chosen for the analysis (Table 2). Spring images were more often subjected to snow, clouds, and other atmospheric disturbances, which meant that more images were needed to obtain representative data.

#### NDVI composite calculations

NDVI was calculated for all images based on the red and near infrared bands. For each period two NDVI composites were calculated, one pre-harvest and one post-harvest. Maximum value composites (MVC’s) of NDVI for pre-harvest were computed to capture the greenest pixels of the period (i.e., actively cultivated pixels). For post-harvest representation, median value composites were calculated for all periods. To better capture harvested areas, median was used instead of maximum, since maximum would include fallow areas with some dense natural vegetation and because minimum values would include bad pixels affected by e.g., clouds. Composites based on multi-year stacks help decrease the influence of seasonal fluctuations in precipitation, clouds, and atmospheric disturbances and also agricultural fallow practices (Holben 1986).

#### Segmentation

One scene for each period (Table 2) was used in a segmentation in order to merge pixels with similar characteristics into vector objects (polygons) to allow for an object-based analysis using the eCognition software (Trimble 2012). The segmentation method used was “multiresolution” that uses the reflectance values of the satellite images seeking to minimize the heterogeneity of the objects with regards to both object shape and spectral properties (Darwish et al. 2003; Trimble 2012). The segmentation used the bands green, red, and NIR (with equal weights) but excluded the blue band to avoid influence of haze, common in these areas. Different settings for the scale parameter (100), shape (0.1), and compactness (0.1) were tested and the combination that appeared to provide the best result (based on a visual inspection) was used. Each object was assigned an NDVI difference value averaged over the polygon area. For all periods, all segments with their centroid in a water body were masked.

#### Determining threshold values

Training data were used to determine threshold values to help classify active winter cropland and were sampled from Quickbird (2.4 m), Worldview (1.84 m), and SPOT (2.5–10 m) provided by Google Earth through the QGIS OpenLayers plugin (Sourcepole 2013) for the present period (2011–2014). Based on the 333 training data points (of which 51 were cropland) and the NDVI difference data for the present period (D), statistics such as mean, standard deviation, and median were computed for both cropland and other land covers (including bare soil, built-up area, orchard, grassland, shrub, and woodland). For cropland, the mean difference between summer and spring was 0.5713. The mean value for cropland minus 2.3 standard deviations (0.5713 − 2.3 × 0.113 = 0.30291) was determined as a lower threshold to separate winter crops from other land uses. This theoretically included close to 99 % of the population if normally distributed, and was the threshold that overall led to the most accurate classification for all periods.

All segments with an average mean NDVI range larger than, or equal to, 0.30291 were classified as active winter cropland. Due to the overlap in phenology between grassland and winter cropland, grasslands were masked by using topographic data from the ASTER digital elevation model (ASTER 2011). A slope image was calculated and mean slope values for each segment were extracted. A threshold was defined based on the training data that showed that cropland is substantially different in slope from the other classes. Slope values ≥7.625° (mean value for cropland + two standard deviations) were filtered from the cropland layer. All four periods’ polygons classified as winter crops were combined in an overlay to show areas that had been cultivated throughout all four periods and areas that had only been cultivated during some of the periods.

#### Validation

Ground Truth Points were collected during a fieldwork in the Duhok governorate in 2013 and used for validation of the classification for period D. These points were sampled with a car collecting data every km on the right hand side of the road resulting in 240 ground truth points. For the remaining periods, a visual classification of the same 240 point locations and their corresponding polygon segments were based on bands 2, 3, and 4 of the spring period (Olofsson et al. 2014). An accuracy assessment was performed on all periods where the classification results were compared to the validation data. User’s and producer’s accuracies and confidence intervals (at the 95 % level) of area estimates were calculated for active winter cropland and other land covers (i.e., 2 classes) using the AccTool (Olofsson et al. 2013).

## Results

### Changes in winter cropland

**Fig. 2:**
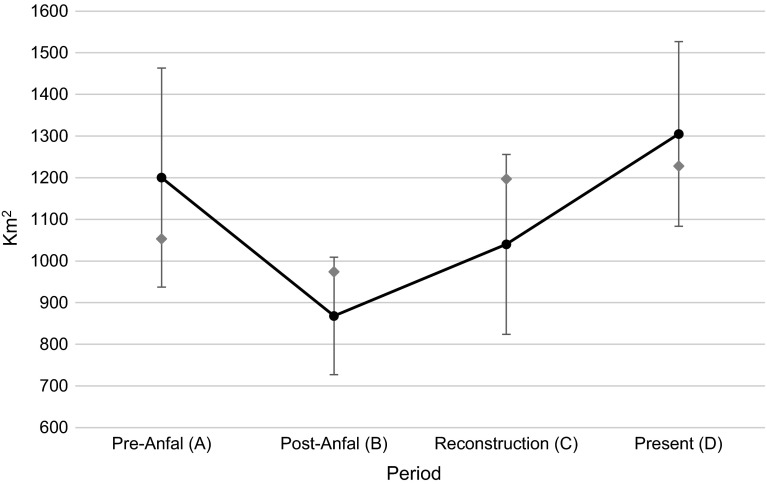
The adjusted area estimates (*black markers*) of winter crops within the Duhok governorate throughout the four periods with confidence intervals (at the 95 % level). The *gray markers* represent the area of cropland before calculating adjusted area estimates

**Fig. 3:**
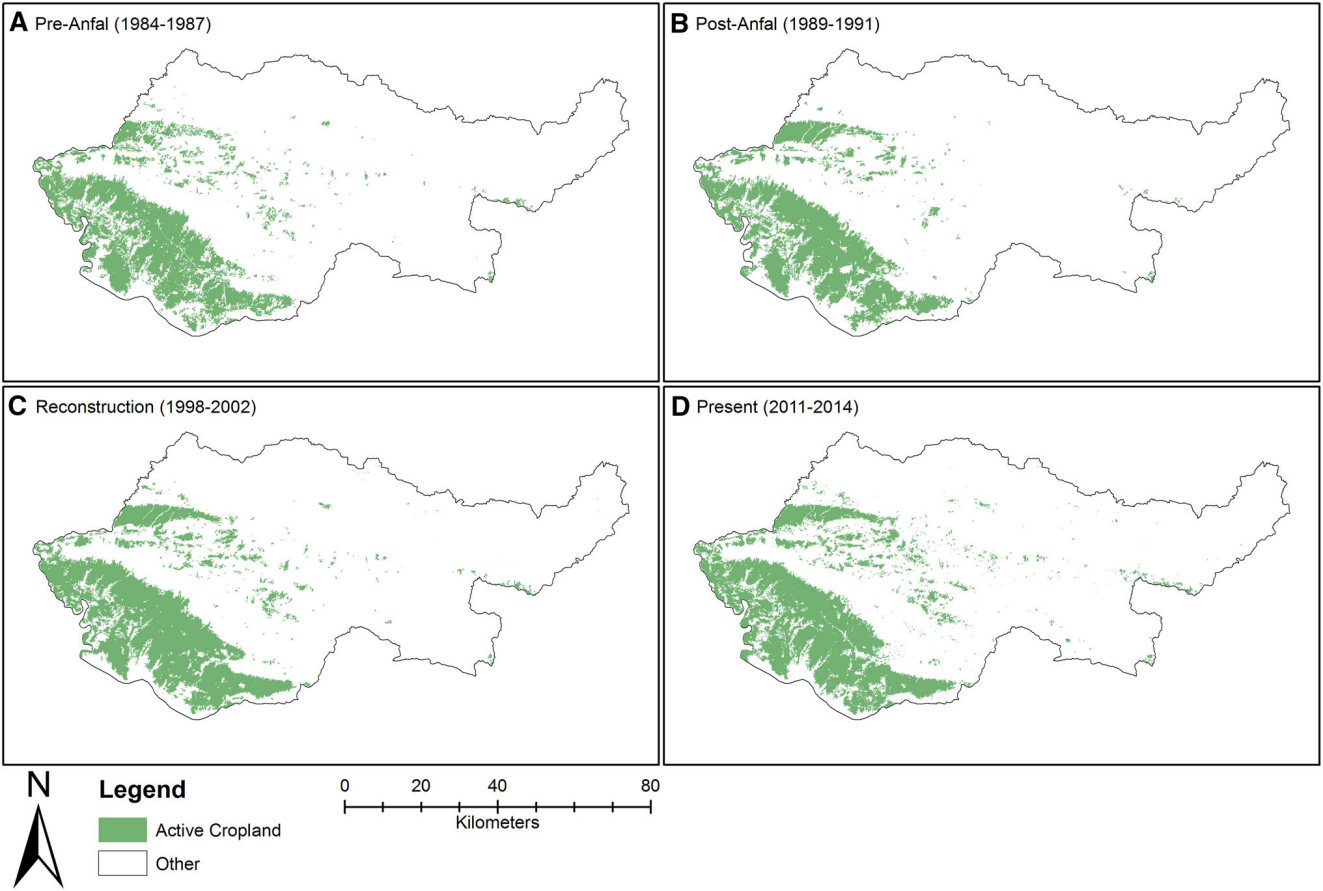
Areas classified as active cropland during the four periods

This analysis shows that an estimated 1200 (±263) km^2^ of the Duhok governorate area were cultivated with winter crops before the *Anfal* campaign (Fig. 2). In the post-*Anfal* period, the estimated area had seen a potential decrease to 868 (±141) km^2^, but then a slight increase to 1040 (±216) km^2^ in the reconstruction period. A potential increase can be seen in the present period where 1300 (±222) km^2^ were cultivated with cropland. Most winter crop agriculture is located in the south western parts of the governorate, in lower elevation areas (Fig. 3).

**Fig. 4:**
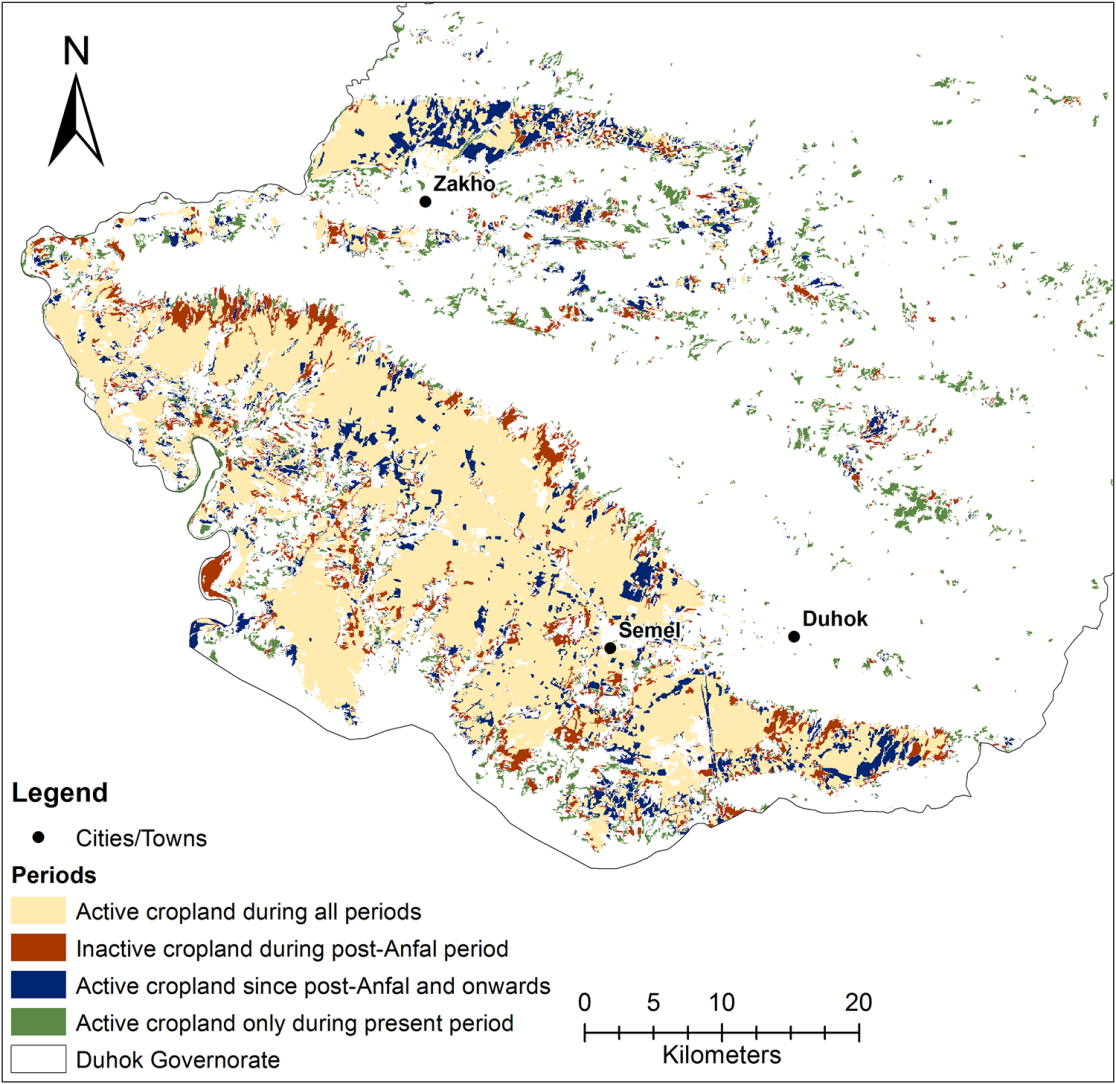
Winter crop changes in the southwestern part of the Duhok government. Note that only combinations of periods >100 km^2^ were included

The overlay analysis showed that about 660 km^2^ (10 % of the Duhok governorate area) had been actively cultivated with winter crops throughout all four periods (Fig. 4). These stable croplands were mainly located in the south western part of the Duhok governorate and north of Zakho city (Fig. 4). Another 125 km^2^ (1.9 %) was consecutively cultivated in the three periods after *Anfal*, i.e., periods B, C, and D. An area of 115 km^2^ (1.8 %) was cultivated in all periods but the post-*Anfal* period (B). An expansion of 114 km^2^ (1.8 %) cropland onto land previously not used for winter crops was seen during the present period (D). Areas partly cultivated throughout the period of interest were located both in proximity to the consecutively cultivated area in the south west, and also in the central parts of the Duhok governorate (Fig. 4). A total of approximately 1500 km^2^ (23 % of the whole Duhok governorate area) were during one or more periods cultivated with winter crops.

### Classification accuracy

The accuracy assessment showed user accuracies of between 69.2 and 96.6 % for the active cropland class, and producer accuracies of between 61.2 and 93.1 % (Table 3). For the “Other Land Covers” class user accuracies ranged between 91.4 and 96.3 % and producer accuracies between 91.6 and 98.9 %. Overall accuracies were between 93.4 and 97.1 % and kappa values between 0.6 and 0.86.

**Table 3:** Error matrices and accuracy assessment results for the classifications

Classification	Truth			
	Agriculture	Other	Classification overall	Producer accuracy
Period A				
Agriculture	30	13	43	61.2 %
Other	16	174	190	94.1 %
Truth overall	46	187	233	
User accuracy	69.8 %	91.6 %		
Overall accuracy	88.1 %		Kappa	0.597
Period B				
Agriculture	34	7	41	93.1 %
Other	2	184	186	97.1 %
Truth overall	36	191	227	
User accuracy	82.9 %	98.9 %		
Overall accuracy	96.6 %		Kappa	0.859
Period C				
Agriculture	36	16	52	79.7 %
Other	7	171	178	93.4 %
Truth overall	43	187	230	
User accuracy	69.2 %	96.1 %		
Overall accuracy	91.2 %		Kappa	0.696
Period D				
Agriculture	38	8	46	77.7 %
Other	10	174	184	96.0 %
Truth overall	48	182	230	
User accuracy	82.6 %	94.6 %		
Overall accuracy	92.3 %		Kappa	0.759

## Discussion

### Cropland area changes

The results shed new light on the effects of the *Anfal* attacks on land cover in Duhok, as they partly refute the findings of Mubareka and Ehrlich (2010) that no post-*Anfal* land use change was detected in the Duhok governorate. The differing results may be explained by the fact that we look at the whole Duhok governorate, and therefore, are able to see more overall changes. On the other hand the changes are not as strong as the ones found in the Jafati valley. Thereby our results add value to qualitative reports such as those of Human Rights Watch (1993) and Hardi (2011) who are stating that *Anfal* impacted agriculture, by providing an indication of the extent and location of land change. We also take a step further and analyze the recovery of agriculture after *Anfal*, during the village reconstruction program, as well as a decade later, during the strong economic development in the KR-I. The spatial assessment gives insights about, for example, areas that experienced agricultural inactivity after *Anfal* and which areas that were consecutively cultivated during all periods.

The results of this analysis show a declining trend in winter crop area after the *Anfal* attack in 1988, when many people were killed or forcibly moved and about 2000 villages in KR-I were destroyed (Human Rights Watch 1993; Mubareka and Ehrlich 2010). Between the pre-*Anfal* period (A) and the post-*Anfal* period, the adjusted cropland area estimates changed from about 1200 to <900 km^2^, showing a decreasing trend of about 400 km^2^. The confidence intervals of these estimates, however, have a range of ±263 and ±141 km^2^, respectively, which means that the change might be smaller or larger due to potential over- and under-classification errors. The overlap between the confidence intervals between period A and B is rather small (72 km^2^) and a decrease in cropland of up to 736 km^2^ is possible. This overall change in the Duhok governorate is also supported by the more detailed analysis at segment level. About 115 km^2^ of land had been used for winter crops during all periods but the post-*Anfal* period, indicating land abandonment during *Anfal* and then a return during the reconstruction program. However, despite the overall decrease in winter cropland during this period (B) 125 km^2^ that were previously un-cultivated were converted to winter crop lands. This could indicate a displacement of agricultural activity to new areas within the Duhok governorate similar to what Baumann et al. (2014) found in their study of the Nagorno-Karabakh conflict.

The village reconstruction program initiated in 1991 intended to help people return to and rebuild their homes and lands. The findings of Eklund and Pilesjö (2012), reporting that many people had returned from urban areas to villages, are likely related to this reconstruction program. The adjusted area estimates for cropland show that during the latter part of this program (1998–2002, period C), the area of cultivated winter crops had increased slightly, but the confidence intervals show a large overlap between these two periods, which means that the changes could have been both positive and negative, with an increase of up to 529 km^2^ or a decrease of down to 185 km^2^. Some 115 km^2^ of winter cropland that had been inactive during the post-*Anfal* period (B) were reclaimed during the reconstruction program and continued to be cultivated in the present period (D). This indicates that the reconstruction program might have led to increased winter crop activity by reclaiming and extending agricultural lands. This period also coincided with the international and national trade sanctions, and with the Oil-for-Food programme, which reportedly impacted the agricultural development negatively. The sanctions reduced the ability to import agricultural input products and infrastructure material that could help an intensification of agriculture (which might be one reason behind the extension in cultivated areas), while the Oil-for-Food Programme created a dependency on food aid and reduced the need for agricultural production.

After the reconstruction period (C) the adjusted area estimates of winter crop land saw a further increase, from 1040 (±216) km^2^ to 1300 (±222) km^2^. Between periods C and D, there is a confidence interval overlap of 173 km^2^ (representing a potential decrease), but the increase might be as large as 481 km^2^. During this period (D), around 115 km^2^ of previously inactive land was converted to winter crop land. Since 2003, after the fall of the Iraqi regime led by Saddam Hussein, the KR-I has developed its economy rapidly, with increased foreign investments, oil exploration, and even tourism. This type of economic development is often accompanied by a rapid urbanization due to increased economic opportunities in cities (Tolley 1987). Field work indicated that many people who had returned to rural areas since the reconstruction program were eager to move back to the urban areas, mainly for better education and health care services, but that they were not able to do so due to lack of affordable housing options. This period might also have seen an intensification of agriculture by for example irrigation and fertilization, which was possible since 2003 when sanctions were lifted and imports resumed.

### Reflections on data and methods

Using crop phenology of multi-temporal, medium resolution satellite images is a useful approach for areas and periods where agriculture is small scale and data are scarce. These types of analyses, however, require contextual knowledge about the study area, for example sowing and harvest information and political situations, in order to interpret the results.

The threshold value used to distinguish between winter crops and other land uses determines if areas will or will not be classified as agricultural land. This may lead to the inclusion of other land-use types, and exclusion of winter crop land in some cases, leaving some uncertainties.

The accuracy assessment showed generally high accuracies for both active cropland and other land covers. Periods A–C, validated through visual interpretation of spring images, had accuracies similar to Period D, validated with ground data from the field. The user’s accuracy, representing to what extent the classification can be expected to reflect reality, for active winter cropland shows that between 69 and 83 % of the classifications were correct. The producer’s accuracy, representing how well the winter crop areas have been classified, shows that between 61 and 93 % of the areas that were winter crops in the validation data were also classified as winter crops. Due to these varying classification accuracies, however, some caution should be taken when talking about absolute changes in winter crop area. The adjusted area estimates presented in Fig. 2 show a trend that corresponds well with the political and socio-economic contexts of the four periods. Whereas the error bars at 95 % confidence level indicate large uncertainties in the estimated cropland changes, these uncertainties would be lower at a confidence level of e.g., 90 %.

While it is possible to calculate uncertainties in the overall area estimates it is difficult to identify where these errors are located and hence where the spatial assessment of cropland changes show inaccuracies. Areas identified as change hot-spots should therefore be further investigated by using both quantitative and qualitative methods at a more local level.

Precipitation variability between years and months influences the vegetation greenness and thus the NDVI values. This influence is, however, accounted for by using composites instead of 1-day images and by focusing on the relative change in NDVI between spring and summer.

## Conclusions

Three political and socio-economic events that have shaped the migration patterns in Kurdistan since the mid-1980s can be identified in the literature: Displacement during the *Anfal* campaign in 1988, return migration between 1991 and 2003, and a new urbanization since the fall of the Iraqi regime in 2003. This study highlights the connection between such events and changes in the agricultural landscape.

Reports of a reduction in agricultural activity after the *Anfal* campaign are supported and clarified by the findings in this paper, showing a likely decrease of about 400 km^2^ in winter crop land. During the reconstruction period (C) slightly more winter cropland were classified as active compared to the post-*Anfal* period. This shows that some land that were abandoned after *Anfal* were reclaimed during the reconstruction period. Due to trade sanctions during this period (C), intensification of agriculture was not possible, which could explain why winter cropland was extended. Ten years after the end of the reconstruction period and the fall of the Iraqi regime, during the present period (D), an increase of approximately 300 km^2^ in estimated winter cropland was recorded, and some previously un-cultivated areas were converted to winter croplands.

The disturbances during 2014, with the conflict between the autonomous organization Islamic State (IS) and the Kurdish and Iraqi governments, show that this region is still shaped by conflicts. In this paper, a simple method of tracking conflict-related population-environment issues quantitatively, with a high accuracy, is provided to support qualitative reports. This method, however, is based on contextual knowledge, so whereas the general idea can be used for any region throughout the world, the analysis needs to be accustomed to the settings of a particular study area, using both quantitative training data and qualitative information to interpret the results.
